# Effects of Metformin Treatment on Liver Glucose Metabolism-Related Enzyme Levels in Streptozotocin-Induced Diabetic Rats

**DOI:** 10.3390/jcm15208108

**Published:** 2026-10-19

**Authors:** Cihan Gül, Mustafa Kelle, Hacer Kayhan Kaya, Bahattin Bulduk, Yılmaz Koçak, Nizamettin Günbatar, İzzet Çeleğen

**Affiliations:** 1 Department of Physiotherapy and Rehabilitation, Faculty of Health Sciences, Van Yüzüncü Yıl University, 65080 Van, Turkey; 2 Department of Physiology, Faculty of Medicine, Dicle University, 21000 Diyarbakır, Turkey; 3 Türkoğlu District Health Directorate, Republic of Turkey Ministry of Health, 46800 Kahramanmaraş, Turkey

**Keywords:** diabetes mellitus, liver enzymes, metformin, glucose metabolism, streptozotocin

## Abstract

**Background:** Diabetes mellitus (DM) disrupts hepatic glucose handling and alters pathways involved in glycogen metabolism, glycolysis, the pentose phosphate pathway, and gluconeogenesis. This study examined hepatic glycogen content and glucose metabolism-related enzyme levels at defined stages of streptozotocin (STZ)-induced diabetes and evaluated the effect of metformin at week 8. **Methods:** Sixty-three adult male Wistar Albino rats were initially allocated to nine groups (*n* = 7/group): four time-matched control groups (C1–C4), four untreated diabetic groups (D1–D4), and one metformin-treated diabetic group (T). Diabetes was induced with a single intraperitoneal dose of STZ (35 mg/kg). D1/C1, D2/C2, D3/C3, and D4/C4 were evaluated at weeks 1, 3, 6, and 8, respectively; T received metformin (100 mg/kg/day) in drinking water and was evaluated at week 8. One animal each in D3, D4, and T died spontaneously before the scheduled endpoint, leaving 60 animals for final analyses. Hepatic glycogen and glucose metabolism-related enzyme levels were measured by rat-specific sandwich ELISA. **Results:** Time-matched comparisons showed significant diabetes-associated differences in hepatic glycogen and all measured enzyme levels at weeks 1, 3, 6, and 8. At week 8, metformin shifted glycogen, glycogen synthase (GS), glycogen phosphorylase (GP), lactate dehydrogenase (LDH), pyruvate kinase (PK), hexokinase (HK), glucose-6-phosphatase (G6Pase), glucose-6-phosphate dehydrogenase (G6PD), and fructose-1,6-bisphosphatase (FBP1) toward control values compared with untreated diabetic rats (Holm-adjusted *p* = 0.0035 for each comparison). However, T remained significantly different from C4 for every hepatic parameter, and final fasting blood glucose did not differ significantly between T and D4. **Conclusions:** STZ-induced diabetes was associated with marked alterations in hepatic glycogen and glucose metabolism-related enzyme levels. Metformin partially ameliorated these hepatic changes at week 8 despite persistent severe hyperglycemia, but it did not normalize the measured hepatic profile.

## 1. Introduction

Diabetes mellitus (DM) remains a rapidly escalating global health challenge with profound clinical and socioeconomic implications. According to the National Diabetes Statistics Report, over 422 million individuals were affected by type 2 diabetes globally in recent years, with approximately 30.3 million cases in the United States alone [1]. The clinical burden is further exacerbated by nearly 7.2 million undiagnosed cases and 86.1 million individuals with prediabetes, a condition characterized by elevated glycemic levels that significantly elevate the risk of progression to overt type 2 diabetes [1]. The chronicity of DM is associated with severe microvascular and macrovascular complications, among which diabetic nephropathy stands out as a life-threatening manifestation [2]. Despite advancements in therapeutic interventions, the complex pathophysiology of diabetes—characterized by chronic hyperglycemia and profound disruptions in carbohydrate, lipid, and protein metabolism—continues to cause multi-organ damage, particularly in the cardiovascular and nervous systems [3].

As a metabolic disorder, DM arises from absolute insulin deficiency or impaired insulin action, leading to systemic abnormalities in carbohydrate processing [4]. The physiological attempts to compensate for these metabolic failures impose an immense burden on the endocrine system, resulting in a breakdown of homeostatic control [5]. This endocrine dysregulation directly alters the activity of key glucose metabolic enzymes, fundamentally driving the onset and maintenance of persistent hyperglycemia.

The liver is the central orchestrator of glucose homeostasis, balancing glucose storage and production according to metabolic demand [6]. During fasting, endogenous glucose production is supplied predominantly by the liver through both glycogenolysis and gluconeogenesis, while the kidney also contributes through gluconeogenesis; the relative contribution of these pathways varies with fasting duration and metabolic conditions [6,7]. In the diabetic state, this delicate balance is disrupted. Specifically, the activities of glucokinase and glucose-6-phosphatase (G6Pase) serve as critical determinants of net glucose flux. Evidence suggests that hepatic glucokinase activity significantly declines in diabetes, whereas G6Pase level can increase nearly twofold, favoring excessive glucose output [8].

Increased hepatic gluconeogenesis and glycogenolysis are core hallmarks of the diabetic state and primary drivers of fasting hyperglycemia [9]. Given that insulin is a principal regulator of these metabolic pathways, experimental models of diabetes demonstrate alterations in hepatic carbohydrate-metabolizing enzymes and glucose metabolism [10].

Metformin is a widely used first-line pharmacological treatment for type 2 diabetes and lowers glycemia primarily by reducing hepatic glucose production while also influencing peripheral glucose handling [11,12,13]. Its hepatic actions involve multiple mechanisms affecting gluconeogenesis and cellular energy metabolism [12,13]. Metformin has also been associated with modest improvements in lipid parameters, particularly total and low-density lipoprotein cholesterol [14].

Despite our extensive knowledge of metformin’s clinical benefits, the time-dependent changes in specific hepatic metabolic enzymes during the progression of diabetes—and the degree to which metformin can preserve these enzymatic profiles—require further elucidation. In this study, we aimed to evaluate these temporal enzymatic shifts in a streptozotocin-induced diabetic model.

## 2. Materials and Methods

### 2.1. Ethical Approval

This study was conducted at the Dicle University Health Sciences Application and Research Center following approval from the Dicle University Animal Ethics Committee (Decision No: E-35582840-020-494517, Date: 15 May 2023). All procedures were performed in accordance with the “Guide for the Care and Use of Laboratory Animals” and international ethical guidelines.

### 2.2. Animals and Environmental Conditions

Sixty-three adult male Wistar Albino rats (age: 8–10 weeks; weight range: 300–490 g, selected to ensure physiological maturity for long-term progressive metabolic tracking) were obtained from the Prof. Dr. Sabahattin Payzın Health Sciences Research and Application Center, Dicle University. The animals were housed in a controlled environment with a temperature of 22 ± 2 °C, 65% ± 5% humidity, and a 12-h light/dark cycle. Rats were provided with a standard pellet diet and tap water ad libitum. Strict inclusion criteria and randomized distribution were applied across all experimental cohorts to minimize baseline physiological variations.

### 2.3. Induction of Experimental Diabetes

Diabetes was induced by a single intraperitoneal (i.p.) injection of streptozotocin (STZ; Sigma-Aldrich, St. Louis, MO, USA) at 35 mg/kg, freshly dissolved in 0.1 M citrate buffer (pH 4.5). Single-dose intraperitoneal STZ protocols in Wistar rats have used doses including 35 mg/kg, and practical guidance describes 35–65 mg/kg as a commonly used diabetogenic range in rats; dose selection should balance successful diabetes induction against excessive toxicity and mortality [15,16]. Control rats received an equivalent volume of citrate buffer. To reduce the risk of early post-STZ hypoglycemia, 10% glucose was provided in drinking water for the first 24 h. Fasting blood glucose was measured 72 h after STZ administration, and rats with values ≥ 250 mg/dL were considered diabetic and included in the diabetic groups.

### 2.4. Experimental Design and Treatment Protocol

The rats were randomly allocated to nine experimental groups (*n* = 7 per group initially). C1, C2, C3, and C4 were non-diabetic controls evaluated at weeks 1, 3, 6, and 8, respectively. D1, D2, D3, and D4 were untreated diabetic groups evaluated at the corresponding time points. The T group consisted of diabetic rats treated with metformin (100 mg/kg/day) in drinking water and evaluated at week 8. The 1-, 3-, 6-, and 8-week endpoints were selected a priori to provide cross-sectional snapshots of early, intermediate, and later metabolic changes during sustained diabetes. Week 8 was defined as the final observation point so that the cumulative effect of metformin treatment could be evaluated directly against the time-matched untreated diabetic (D4) and control (C4) groups. Each terminal time point therefore represented an independent cohort rather than repeated measurements in the same animals.

Rats were group-housed, and individual animals were identified by numbers marked on their tails. Water consumption was monitored at the cage level. Therefore, although the metformin concentration in drinking water was adjusted weekly to target a dose of 100 mg/kg/day, the exact metformin intake of each individual rat could not be directly quantified.

A total of 63 rats entered the experiment. During follow-up, one animal each in D3, D4, and T died spontaneously before the scheduled endpoint. No protocol-triggered early euthanasia was documented in the study records; the three losses were spontaneous deaths before the planned sacrifice time points. Accordingly, the final analyses included 60 animals: D1 = 7, C1 = 7, D2 = 7, C2 = 7, D3 = 6, C3 = 7, D4 = 6, C4 = 7, and T = 6. Severe hyperglycemia and weight loss were observed during follow-up and are reported as relevant adverse clinical findings (Table 1).

### 2.5. Tissue Collection and Preparation

At the end of their respective experimental periods (Weeks 1, 3, 6, or 8), animals were fasted overnight and sacrificed under anesthesia via cardiac puncture. Liver tissues were rapidly excised, rinsed in ice-cold saline to remove residual blood, and stored at −80 °C for further biochemical analysis.

### 2.6. Biochemical Analyses

Hepatic glycogen content and glucose metabolism-related enzyme levels were quantified using rat-specific sandwich enzyme-linked immunosorbent assay (ELISA) kits (SunRed Biological Technology, Shanghai, China). The measured proteins were hexokinase (HK), pyruvate kinase (PK), glucose-6-phosphatase (G6Pase), fructose-1,6-bisphosphatase (FBP1), lactate dehydrogenase (LDH), glycogen synthase (GS), glycogen phosphorylase (GP), and glucose-6-phosphate dehydrogenase (G6PD). Enzyme levels were calculated from the kit-specific standard curves and are reported in the original assay units: ng/mL for HK, PK, G6Pase, FBP1, GS, GP, and G6PD, and U/L for LDH. Total protein content in liver homogenates was determined by the Lowry method using bovine serum albumin as the standard; however, the ELISA-derived enzyme values reported here were not converted to µmol/mg protein. Accordingly, the results are referred to throughout the manuscript as enzyme levels rather than enzyme activities.

### 2.7. Statistical Analysis

Statistical analyses were performed using SPSS version 26.0. Data are presented as mean ± standard deviation (SD). Because terminal hepatic measurements were obtained from different animals at each scheduled endpoint, these data were treated as independent, time-matched cohorts rather than repeated measures. At weeks 1, 3, and 6, diabetic and control groups were compared using two-sided Mann–Whitney U tests (D1 vs. C1, D2 vs. C2, and D3 vs. C3). At week 8, C4, D4, and T were compared using the Kruskal–Wallis test followed by pairwise comparisons with Holm adjustment. Final body weight and final fasting blood glucose were analyzed using the same time-matched strategy. Food and water intake were summarized descriptively because they were recorded at cage level. Pearson correlation and linear regression were used to examine relationships between hepatic glycogen and GS or GP levels in pooled diabetic and pooled control animals. Two-sided *p* < 0.05 was considered statistically significant.

## 3. Results

### 3.1. Effect of Metformin on Body Weight, Blood Glucose, Food, and Water Intake

Body weight, fasting blood glucose, and recorded food and water intake are summarized in Table 2.

At week 1, final body weight did not differ significantly between D1 and C1 (*p* = 0.710), whereas final fasting blood glucose was higher in D1 than in C1 (*p* = 0.0023). At weeks 3 and 6, diabetic rats had lower final body weight and higher final fasting blood glucose than their corresponding controls (D2 vs. C2: *p* < 0.001 for both; D3 vs. C3: *p* = 0.0012 for both).

At week 8, overall differences among D4, C4, and T were significant for final body weight (Kruskal–Wallis *p* = 0.0011) and final fasting blood glucose (*p* = 0.0017). D4 and T each differed from C4 for both outcomes (Holm-adjusted *p* = 0.0035), whereas D4 and T did not differ significantly from each other (final body weight, adjusted *p* = 0.132; final fasting blood glucose, adjusted *p* = 0.589). Thus, metformin did not significantly improve final body weight or fasting glycemia relative to untreated diabetic rats at week 8.

Food and water intake showed higher descriptive values in diabetic groups than in time-matched controls. At week 8, T showed lower recorded food and water intake than D4 but remained above C4. These cage-level measurements were not subjected to animal-level inferential testing.

### 3.2. Effect of Metformin on Hepatic Glucose Metabolism and Enzyme Levels

Time-matched comparisons showed marked diabetes-associated alterations in hepatic glycogen content and glucose metabolism-related enzyme levels (Table 3). At weeks 1 and 3, all nine hepatic parameters differed between diabetic rats and their corresponding controls (D1 vs. C1 and D2 vs. C2; *p* < 0.001 for all comparisons). At week 6, all parameters also differed between D3 and C3 (*p* < 0.01 for all comparisons).

Relative to the corresponding time-matched controls, diabetic groups showed lower hepatic glycogen, glycogen synthase (GS), pyruvate kinase (PK), hexokinase (HK), and glucose-6-phosphate dehydrogenase (G6PD) levels, together with higher glycogen phosphorylase (GP), lactate dehydrogenase (LDH), glucose-6-phosphatase (G6Pase), and fructose-1,6-bisphosphatase (FBP1) levels. This pattern is consistent with reduced glycogen storage and glycolytic capacity together with a shift toward pathways associated with hepatic glucose output. Because different animals were sampled at each endpoint, the across-week pattern is descriptive and should not be interpreted as a within-animal longitudinal trajectory.

At week 8, overall differences among C4, D4, and T were significant for hepatic glycogen and all eight enzyme levels (Kruskal–Wallis *p* < 0.001 for each parameter). Holm-adjusted pairwise comparisons showed significant differences between C4 and D4, D4 and T, and C4 and T for every hepatic parameter (adjusted *p* = 0.0035 for each comparison).

Compared with D4, metformin treatment shifted all measured hepatic parameters toward C4 values: glycogen, GS, PK, HK, and G6PD levels were higher in T, whereas GP, LDH, G6Pase, and FBP1 levels were lower. However, T remained significantly different from C4 for all parameters, indicating partial amelioration rather than normalization.

### 3.3. Correlation Analyses

**Figure 1:**
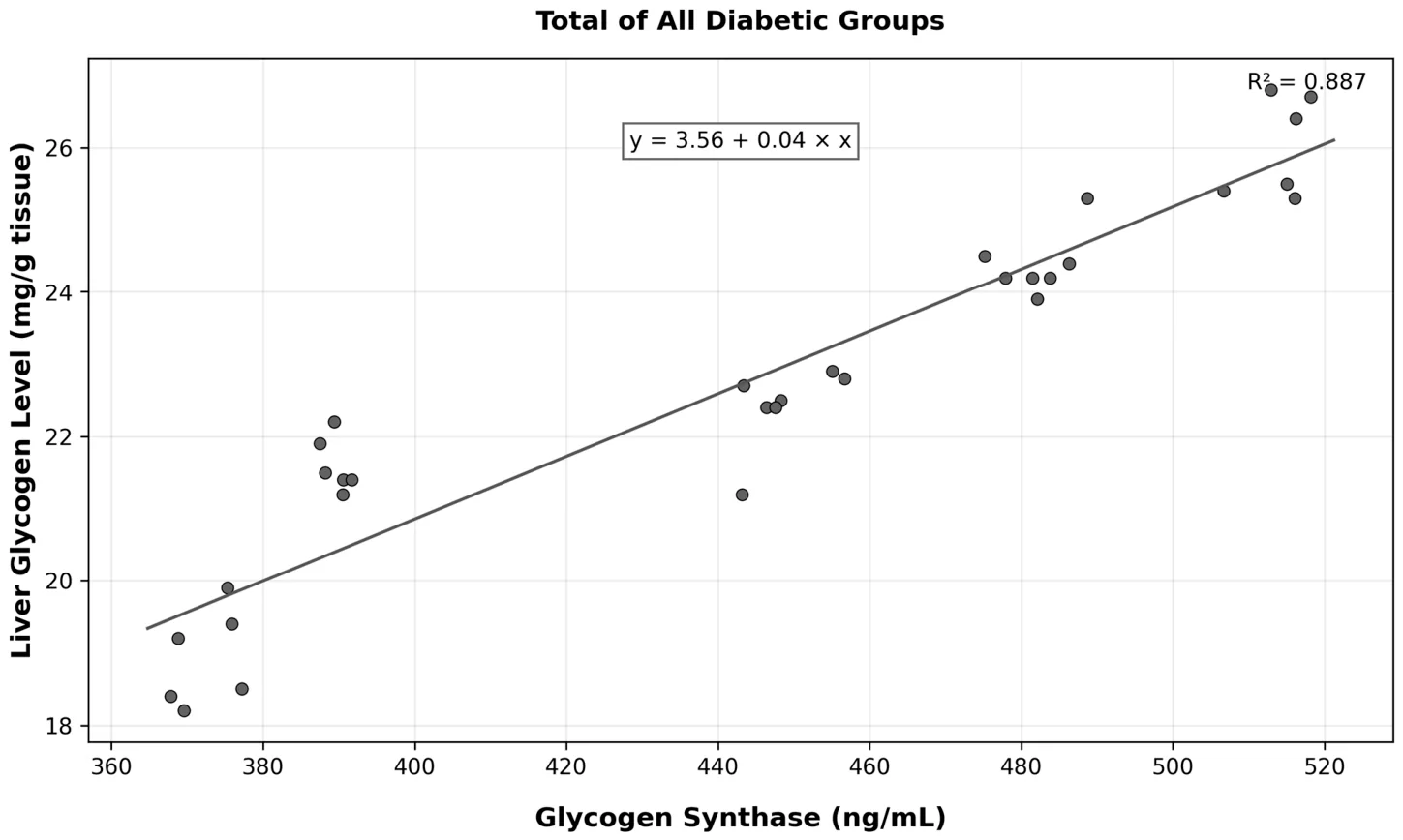
Correlation between Liver Glycogen Level and Glycogen Synthase (GS) in All Diabetic Groups. Legend: Linear regression analysis showing a significant positive association between hepatic glycogen and glycogen synthase (GS) levels across pooled diabetic groups (D1–D4 and T; r = 0.942, R^2^ = 0.887, *p* < 0.001).

**Figure 2:**
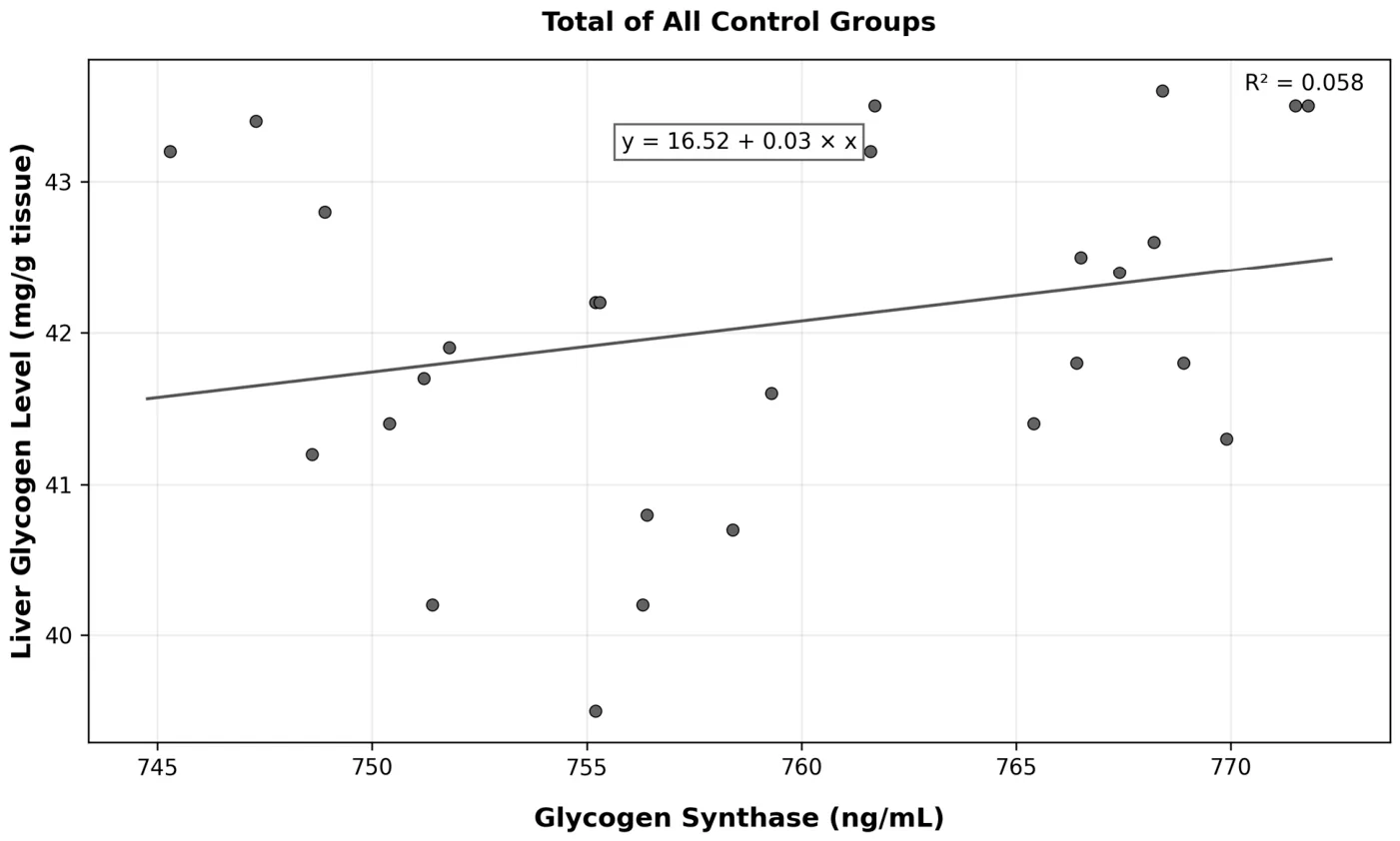
Correlation between Liver Glycogen Level and Glycogen Synthase (GS) in All Control Groups. Legend: Linear regression analysis showing no significant association between hepatic glycogen and glycogen synthase (GS) levels across pooled control groups (C1–C4; r = 0.240, R^2^ = 0.058, *p* = 0.218).

**Figure 3:**
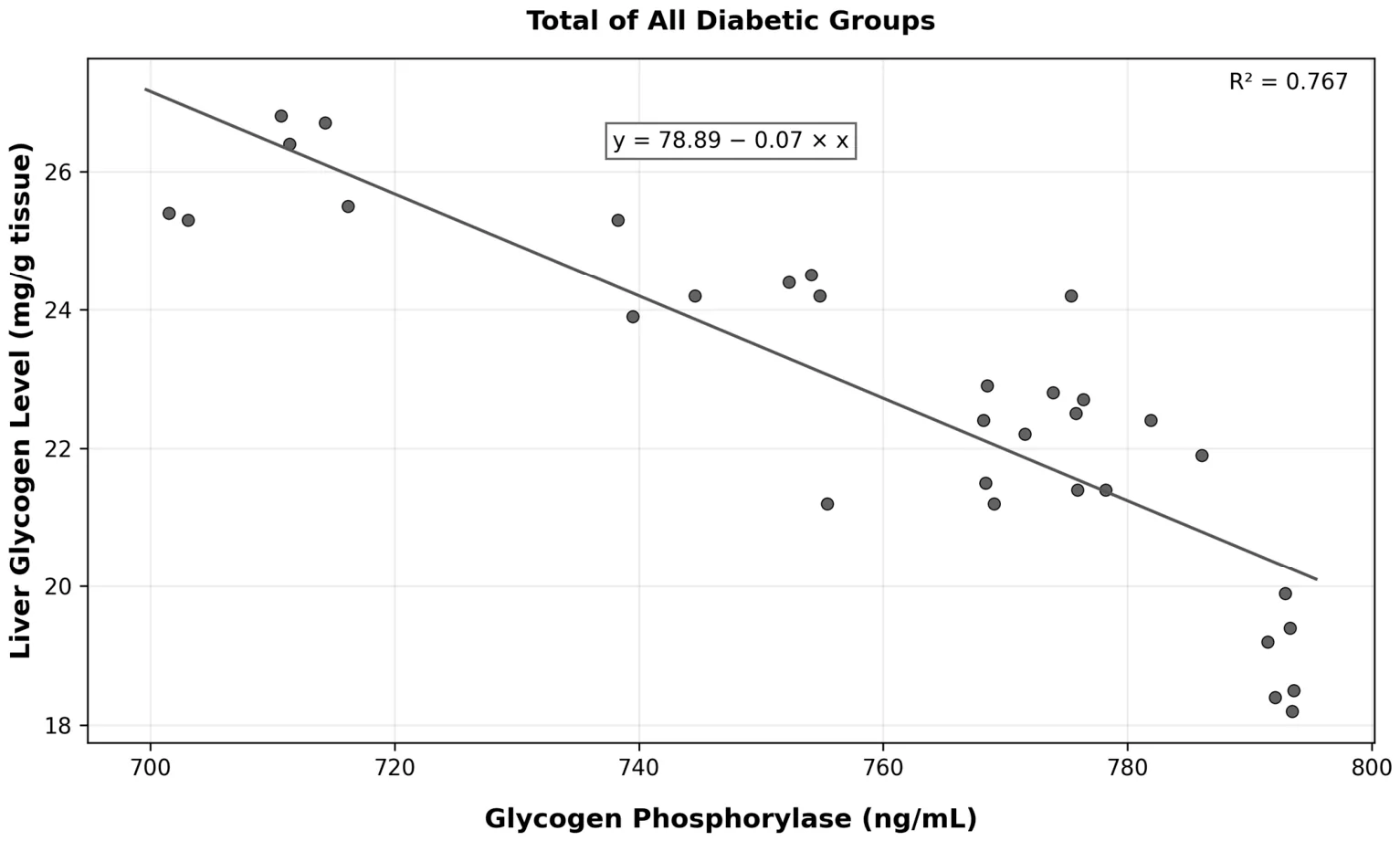
Correlation between Liver Glycogen Level and Glycogen Phosphorylase (GP) in All Diabetic Groups. Legend: Linear regression analysis showing a significant negative association between hepatic glycogen and glycogen phosphorylase (GP) levels across pooled diabetic groups (D1–D4 and T; r = −0.876, R^2^ = 0.767, *p* < 0.001).

**Figure 4:**
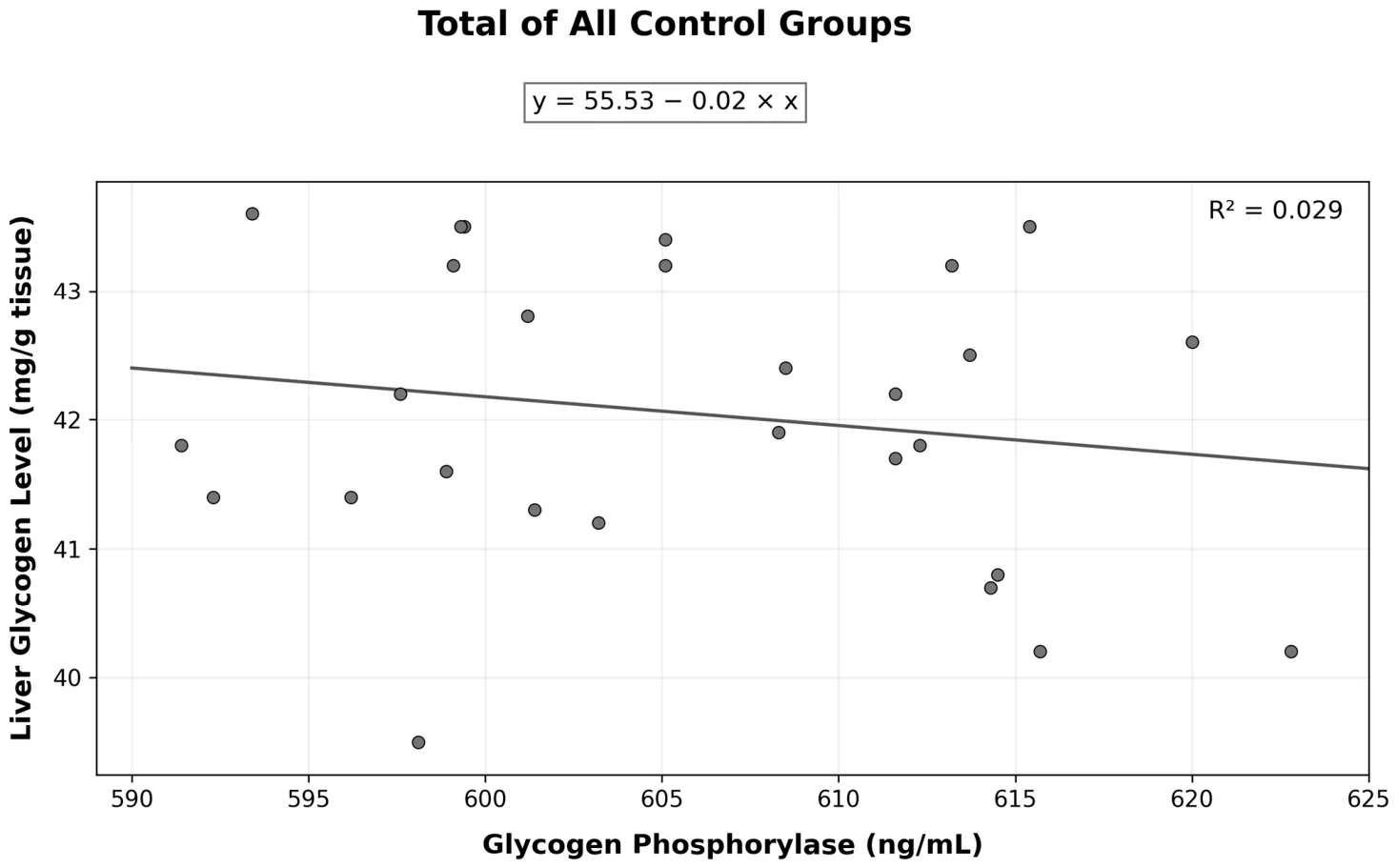
Correlation between Liver Glycogen Level and Glycogen Phosphorylase (GP) in All Control Groups. Legend: Linear regression analysis showing no significant association between hepatic glycogen and glycogen phosphorylase (GP) levels across pooled control groups (C1–C4; r = −0.170, R^2^ = 0.029, *p* = 0.386).

Correlation analyses showed a strong positive association between hepatic glycogen and GS levels in pooled diabetic animals (Pearson r = 0.942, R^2^ = 0.887, *p* < 0.001; Figure 1). In pooled control animals, the glycogen–GS correlation was not significant (r = 0.240, R^2^ = 0.058, *p* = 0.218; Figure 2), A strong negative association was observed between hepatic glycogen and GP levels in pooled diabetic animals (r = −0.876, R^2^ = 0.767, *p* < 0.001; Figure 3). whereas the glycogen–GP correlation was also not significant in pooled control animals (r = −0.170, R^2^ = 0.029, *p* = 0.386; Figure 4).

## 4. Discussion

STZ-induced diabetes produced marked alterations in hepatic glycogen content and glucose metabolism-related enzyme levels. The time-matched analyses consistently separated diabetic animals from their corresponding controls, while the week-8 comparison showed that metformin shifted every measured hepatic parameter toward the control direction without fully normalizing any of them. These findings are consistent with previous reports of marked disturbances in carbohydrate metabolism in experimental STZ-induced diabetes [17]. They should be interpreted as differences in measured enzyme levels rather than direct measurements of catalytic activity.

The physiological data also clarify the limits of the metformin effect in this model. Diabetic animals showed substantial hyperglycemia and lower final body weight at later time points, a pattern compatible with the body-weight loss and enhanced catabolic state described in diabetes [18,19]. At week 8, final fasting blood glucose and body weight did not differ significantly between T and D4. Therefore, the hepatic differences observed with metformin cannot be attributed to demonstrably improved systemic glycemic control in this experiment. Instead, they are consistent with a partial hepatic metabolic effect occurring despite persistent severe hyperglycemia.

Glucokinase is an established regulator of hepatic glucose handling and has been investigated as a therapeutic target in diabetes [20]. However, glucokinase was not measured in the present study; the measured enzyme was hexokinase (HK). This distinction is important when interpreting the present findings.

G6PD levels were reduced in diabetic groups, consistent with disruption of the pentose phosphate pathway reported under diabetic conditions [21,22]. Metformin increased G6PD relative to D4 at week 8, but T remained significantly below C4. Conversely, G6Pase and FBP1 levels were increased in diabetic animals, consistent with enhanced gluconeogenic capacity [23,24,25]. Metformin lowered both proteins relative to D4, but neither reached the corresponding control level. Thus, the data support partial modulation rather than prevention or complete normalization of these diabetes-associated changes.

Hepatic glycogen was lower, GS was reduced, and GP was elevated in diabetic rats, a pattern consistent with impaired glycogen storage and altered glycogen metabolism [26,27]. Metformin partially shifted all three measures toward control values at week 8. The correlation analysis further supported the internal relationship of these measures: hepatic glycogen was strongly positively associated with GS and negatively associated with GP in pooled diabetic animals, whereas neither relationship reached statistical significance in pooled controls.

Hepatic hexokinase (HK) and pyruvate kinase (PK) levels were lower in diabetic animals than in time-matched controls, whereas metformin-treated rats had higher HK and PK levels than D4 but remained below C4. Previous experimental work has documented diabetes-associated alterations in hepatic metabolic enzyme profiles [28]. Related work has also emphasized the cell-specific role of glucokinase in glucose homeostasis [29]; glucokinase, however, is distinct from the HK measured in the present study. In the present study, these findings are compatible with impaired hepatic glucose utilization and a partial metformin-associated shift toward the control profile.

LDH levels were higher in diabetic groups than in time-matched controls and were lower in T than in D4 at week 8, although T remained above C4. LDH catalyzes the interconversion of lactate and pyruvate, but its metabolic effects depend on tissue and isoform context. Structural studies demonstrate isoform-specific properties of LDH [30], while experimental LDH-A overexpression in pancreatic beta cells has been shown to perturb mitochondrial metabolism and insulin secretion [31]. These studies provide biochemical context for LDH but do not establish a liver-specific mechanism for the present findings. Taken together with the other hepatic measurements, our data show a broad but incomplete metformin-associated shift in the hepatic metabolic profile. The absence of a parallel reduction in fasting glycemia in T emphasizes that the present study demonstrates association with hepatic biochemical changes rather than restoration of systemic glucose homeostasis.

The study has several limitations. First, the single-dose STZ model used here produces severe insulin-deficient diabetes and does not reproduce the full pathophysiology of human type 2 diabetes. Second, the ELISA-based measurements quantify enzyme levels rather than catalytic activities; therefore, catalytic function cannot be inferred directly. Third, insulin concentrations and oxidative stress markers were not measured. Fourth, metformin was delivered in drinking water and exposure was estimated from cage-level fluid intake, so individual drug intake could not be verified and inter-animal dose variation cannot be excluded. Fifth, food and water intake were recorded at cage level and were therefore treated descriptively. Finally, different animals were sampled at each terminal time point; consequently, the apparent changes across weeks represent comparisons of independent cohorts rather than repeated measurements within the same animals.

## 5. Conclusions

In this STZ-induced diabetic rat model, hepatic glycogen content and all measured glucose metabolism-related enzyme levels differed substantially from time-matched controls. At week 8, metformin shifted each hepatic parameter toward the control direction compared with untreated diabetic rats, but none was fully normalized, and fasting glycemia remained comparable to D4. These findings indicate partial hepatic metabolic modulation by metformin despite persistent severe hyperglycemia. Further studies incorporating direct enzyme activity assays, insulin measurements, individually quantified drug exposure, and models that more closely reflect insulin-resistant type 2 diabetes are warranted.

## Figures and Tables

**Table 1:** Animal flow summary.

Group	Initial *n*	Spontaneous Deaths	Final *n*
D1	7	0	7
C1	7	0	7
D2	7	0	7
C2	7	0	7
D3	7	1	6
C3	7	0	7
D4	7	1	6
C4	7	0	7
T	7	1	6

**Table 2:** Body weight, water consumption, food consumption and plasma glucose levels of rats.

Groups	*n*	Body Weight (g)		Daily Water Consumption (mL/rat/day)		Daily Food İntake (g/rat/day)		Glucose (mg/dL)	
		Initial	Final	Initial	Final	Initial	Final	Initial	Final
D-1 (7th day)	7	378.29 ± 60.50	375.00 ± 59.63	86.14 ± 1.95	90.57 ± 1.72	31.29 ± 1.11	40.29 ± 1.50	319.00 ± 78.40	344.86 ± 86.96
C-1 (7th day)	7	384.86 ± 41.99	381.86 ± 38.21	82.14 ± 1.07	84.14 ± 2.54	20.43 ± 1.13	23.57 ± 1.81	187.43 ± 38.47	187.14 ± 44.25
D-2 (Week 3)	7	397.86 ± 24.48	331.43 ± 28.33	89.57 ± 1.81	116.86 ± 3.02	30.29 ± 1.80	53.14 ± 1.21	307.71 ± 44.24	385.57 ± 78.30
C-2 (Week 3)	7	450.71 ± 21.68	445.43 ± 21.93	80.86 ± 0.69	81.86 ± 1.35	21.86 ± 1.35	23.14 ± 1.35	104.57 ± 6.63	116.86 ± 7.47
D-3 (Week 6)	6	406.67 ± 47.92	323.67 ± 48.61	89.83 ± 2.14	138.17 ± 1.60	30.17 ± 1.47	63.00 ± 1.79	311.17 ± 66.96	425.83 ± 57.17
C-3 (Week 6)	7	408.71 ± 34.50	411.86 ± 29.64	81.43 ± 0.98	82.14 ± 1.77	21.29 ± 1.38	23.71 ± 1.38	116.00 ± 7.85	103.86 ± 4.63
D-4 (Week 8)	6	404.33 ± 45.55	285.50 ± 37.61	90.00 ± 2.28	180.83 ± 5.78	30.67 ± 1.51	75.67 ± 2.80	273.67 ± 64.74	497.00 ± 60.46
C-4 (Week 8)	7	433.71 ± 24.05	442.14 ± 30.45	80.86 ± 1.77	83.43 ± 1.90	22.29 ± 1.38	23.86 ± 1.21	106.57 ± 6.40	129.57 ± 14.34
T (Week 8)	6	461.17 ± 23.70	317.50 ± 22.90	83.67 ± 2.42	101.50 ± 3.27	23.00 ± 0.89	32.33 ± 2.66	247.83 ± 79.54	513.00 ± 72.63

Abbreviations: D1–D4, diabetic groups; C1–C4, time-matched control groups; T, metformin-treated diabetic group; *n*, number of rats. Data are presented as mean ± SD. For final body weight and fasting blood glucose, D1 vs. C1, D2 vs. C2, and D3 vs. C3 were compared using two-sided Mann–Whitney U tests. At week 8, D4, C4, and T were compared using the Kruskal–Wallis test followed by Holm-adjusted pairwise comparisons. Food and water intake are presented descriptively because these variables were recorded at cage level.

**Table 3:** Hepatic glycogen content and glucose metabolism-related enzyme levels in the experimental groups.

Hepatic Glycogen Content and Glucose Metabolism-Related Enzyme Levels										
Group	*n*	Liver Glycogen (mg/g Tissue)	GS (ng/mL)	GP (ng/mL)	LDH (U/L)	PK (ng/mL)	HK (ng/mL)	G6Pase (ng/mL)	G6PD (ng/mL)	FBP1 (ng/mL)
D-1 (7th day)	7	24.39 ± 0.45	482.21 ± 4.65	751.29 ± 12.62	318.93 ± 2.77	144.64 ± 5.84	134.64 ± 1.87	1773.23 ± 17.62	255.19 ± 4.48	829.97 ± 5.99
C-1 (7th day)	7	42.43 ± 0.99	765.89 ± 5.76	596.93 ± 3.53	253.43 ± 3.85	214.14 ± 2.50	253.14 ± 1.71	964.54 ± 13.67	514.64 ± 9.85	466.79 ± 7.12
D-2 (Week 3)	7	22.41 ± 0.57	448.67 ± 5.32	771.44 ± 8.52	319.17 ± 2.40	125.17 ± 4.26	136.17 ± 2.18	1787.71 ± 17.46	243.29 ± 4.96	835.44 ± 6.48
C-2 (Week 3)	7	42.60 ± 0.58	764.49 ± 6.24	613.53 ± 3.56	248.47 ± 2.81	208.63 ± 2.70	251.64 ± 2.19	955.16 ± 9.87	504.69 ± 9.26	454.44 ± 6.32
D-3 (Week 6)	6	21.60 ± 0.37	389.65 ± 1.59	774.88 ± 6.69	333.58 ± 3.03	119.83 ± 4.08	129.88 ± 1.49	1799.90 ± 12.32	242.08 ± 4.58	837.77 ± 6.48
C-3 (Week 6)	7	42.35 ± 1.13	752.50 ± 6.50	603.90 ± 5.31	249.80 ± 2.22	209.04 ± 3.10	249.84 ± 2.27	948.94 ± 8.51	511.60 ± 8.40	452.96 ± 8.47
D-4 (Week 8)	6	18.93 ± 0.67	372.43 ± 4.14	792.82 ± 0.84	352.33 ± 3.69	108.55 ± 1.38	131.18 ± 1.07	1882.73 ± 7.27	239.97 ± 4.30	841.72 ± 6.75
C-4 (Week 8)	7	40.80 ± 0.89	753.53 ± 3.08	609.01 ± 10.55	253.14 ± 2.55	210.90 ± 1.20	251.39 ± 2.05	956.67 ± 6.66	511.86 ± 8.41	465.93 ± 10.44
T (Week 8)	6	26.02 ± 0.69	514.18 ± 4.05	709.53 ± 5.97	302.00 ± 4.61	139.18 ± 1.97	156.27 ± 2.18	1405.97 ± 14.21	311.38 ± 5.28	782.38 ± 7.57

Statistical analysis: Time-matched comparisons were used. D1 vs. C1, D2 vs. C2, and D3 vs. C3 were compared using two-sided Mann–Whitney U tests. At Week 8, D4, C4, and T were compared using the Kruskal–Wallis test followed by pairwise comparisons with Holm adjustment. Data are presented as mean ± SD.

## Data Availability

The datasets used and analyzed during the current study are available from the corresponding author on reasonable request.

## References

[1] Centers for Disease Control and Prevention (2017). National Diabetes Statistics Report, 2017 Estimates of Diabetes and Its Burden in the United States Background.

[2] Gül C., Kelle M., Baylan M., Yokuş B., Taşdemir E. (2021). Time-dependent changes in plasma ghrelin and angiotensin II levels in the diabetic nephropathy model. Fresenius Environ. Bull..

[3] Vinik A.I., Vinik E. (2003). Prevention of the complications of diabetes. Am. J. Manag. Care.

[4] American Diabetes Association (2010). Diagnosis and classification of diabetes mellitus. Diabetes Care.

[5] Dhananjayan I., Kathiroli S., Subramani S., Veerasamy V. (2017). Ameliorating effect of betanin, a natural chromoalkaloid by modulating hepatic carbohydrate metabolic enzyme activities and glycogen content in streptozotocin—Nicotinamide induced experimental rats. Biomed. Pharmacother..

[6] Roden M., Bernroider E. (2003). Hepatic glucose metabolism in humans—Its role in health and disease. Best Pract. Res. Clin. Endocrinol. Metab..

[7] Alsahli M., Gerich J.E. (2017). Renal glucose metabolism in normal physiological conditions and in diabetes. Diabetes Res. Clin. Pract..

[8] Nordlie R.C., Foster J.D., Lange A.J. (1999). Regulation of glucose production by the liver. Annu. Rev. Nutr..

[9] Punitha I.R., Rajendran K., Shirwaikar A. (2005). Alcoholic stem extract of Coscinium fenestratum regulates carbohydrate metabolism and improves antioxidant status in streptozotocin—Nicotinamide induced diabetic rats. Evid. Based Complement. Altern. Med..

[10] Lv B., Liu S., Li Y., Li Z., An Y., He C., Zhang H., Huang Y., Fu W., Ma Q. (2024). Mulberry leaf ameliorate STZ induced diabetic rat by regulating hepatic glycometabolism and fatty acid β-oxidation. Front. Pharmacol..

[11] Cicero A.F.G., Tartagni E., Ertek S. (2012). Metformin and its clinical use: New insights for an old drug in clinical practice. Arch. Med. Sci..

[12] Czyzyk A., Tawecki J., Sadowski J., Ponikowska I., Szczepanik Z. (1968). Effect of biguanides on intestinal absorption of glucose. Diabetes.

[13] Hermann L.S. (1979). Metformin: A review of its pharmacological properties and therapeutic use. Diabete Metab..

[14] Wulffelé M.G., Kooy A., de Zeeuw D., Stehouwer C.D.A., Gansevoort R.T. (2004). The effect of metformin on blood pressure, plasma cholesterol and triglycerides in type 2 diabetes mellitus: A systematic review. J. Intern. Med..

[15] Ghasemi A., Jeddi S. (2023). Streptozotocin as a tool for induction of rat models of diabetes: A practical guide. EXCLI J..

[16] Mostafavinia A., Amini A., Ghorishi S.K., Pouriran R., Bayat M. (2016). The effects of dosage and the routes of administrations of streptozotocin and alloxan on induction rate of type 1 diabetes mellitus and mortality rate in rats. Lab. Anim. Res..

[17] Shirwaikar A., Rajendran K., Barik R. (2006). Effect of aqueous bark extract of Garuga pinnata Roxb. in streptozotocin-nicotinamide induced type-II diabetes mellitus. J. Ethnopharmacol..

[18] Kalyani R.R., Corriere M., Ferrucci L. (2014). Age-related and disease-related muscle loss: The effect of diabetes, obesity, and other diseases. Lancet Diabetes Endocrinol..

[19] Subash Babu P., Prabuseenivasan S., Ignacimuthu S. (2007). Cinnamaldehyde—A potential antidiabetic agent. Phytomedicine.

[20] Lu M., Li P., Bandyopadhyay G., Lagakos W., DeWolf W.E., Alford T., Chicarelli M.J., Williams L., Anderson D.A., Baer B.R. (2014). Characterization of a novel glucokinase activator in rat and mouse models. PLoS ONE.

[21] Heymann A.D., Cohen Y., Chodick G. (2012). Glucose-6-phosphate dehydrogenase deficiency and type 2 diabetes. Diabetes Care.

[22] Xu Y., Osborne B.W., Stanton R.C. (2005). Diabetes causes inhibition of glucose-6-phosphate dehydrogenase via activation of PKA, which contributes to oxidative stress in rat kidney cortex. Am. J. Physiol. Ren. Physiol..

[23] Bouché C., Serdy S., Kahn C.R., Goldfine A.B. (2004). The cellular fate of glucose and its relevance in type 2 diabetes. Endocr. Rev..

[24] Abdel-Sattar E., EL-Maraghy S.A., El-Dine R.S., Rizk S.M. (2016). Russelioside B, a pregnane glycoside ameliorates hyperglycemia in streptozotocin induced diabetic rats by regulating key enzymes of glucose metabolism. Chem. Biol. Interact..

[25] Zhang Y., Xie Z., Zhou G., Zhang H., Lu J., Zhang W.J. (2010). Fructose-1,6-bisphosphatase regulates glucose-stimulated insulin secretion of mouse pancreatic β-cells. Endocrinology.

[26] Golden S., Wals P.A., Okajima F., Katz J. (1979). Glycogen synthesis by hepatocytes from diabetic rats. Biochem. J..

[27] Dervisevik M., Dinevska-Kovkarovska S., Dimitrovska M., Cipanovska N., Miova B. (2014). High dose of aspirin moderates diabetes-induced changes of heart glycogen/glucose metabolism in rats. J. Physiol. Sci..

[28] Gupta D., Raju J., Prakash J., Baquer N.Z. (1999). Change in the lipid profile, lipogenic and related enzymes in the livers of experimental diabetic rats: Effect of insulin and vanadate. Diabetes Res. Clin. Pract..

[29] Postic C. (2001). Cell-specific roles of glucokinase in glucose homeostasis. Recent. Prog. Horm. Res..

[30] Kavanagh K.L., Elling R.A., Wilson D.K. (2004). Structure of Toxoplasma gondii LDH1: Active-site differences from human lactate dehydrogenases and the structural basis for efficient APAD+ use. Biochemistry.

[31] Ainscow E.K., Zhao C., Rutter G.A. (2000). Acute overexpression of lactate dehydrogenase-A perturbs beta-cell mitochondrial metabolism and insulin secretion. Diabetes.

